# Crystal structure of methyl 2-[2,4-bis­(4-fluoro­phen­yl)-3-aza­bicyclo­[3.3.1]nonan-9-yl­idene]hydrazine­carboxyl­ate

**DOI:** 10.1107/S1600536814018935

**Published:** 2014-08-30

**Authors:** A. Kamaraj, R. Rajkumar, K. Krishnasamy, S. Murugavel

**Affiliations:** a Department of Chemistry, Annamalai University, Annamalainagar 608 002, Chidambaram, Tamilnadu, India; b Department of Physics, Thanthai Periyar Government Institute of Technology, Vellore 632 002, India

**Keywords:** crystal structure, 3-aza­bicyclo­[3.3.1]nona­ne, hydrazine­carboxyl­ate, twin-chair conformation

## Abstract

In the title compound, C_22_H_23_F_2_N_3_O_2_, the bicyclic ring system exists in a twin-chair conformation with an equatorial disposition of the 4-fluoro­phenyl groups on the heterocycle. These aromatic rings are inclined to one another by 19.4 (1)°. In the crystal, mol­ecules are linked by pairs of N—H⋯O and C—H⋯O hydrogen bonds into inversion dimers, incorporating *R*_1_^2^(7) and *R*_2_^2^(8) ring motifs; the same O atom accepts both hydrogen bonds. These dimers are further linked by a pair of C—H⋯F hydrogen bonds, enclosing *R*_2_^2^(28) ring motifs, forming supra­molecular chains along [010]. The NH group of the pyridine ring is not involved in hydrogen bonding, probably due to the steric hindrance of the fluoro­phenyl groups.

## Abstract 2

In the title compound, the bicyclic ring system adopts a twin-chair conformation. In the crystal, N—H⋯O, C—H⋯O and C—H⋯F inter­actions connect the mol­ecules, forming supra­molecular chains propagating along the *b*-axis direction.

## Chemical context   

Mol­ecules containing the 3-aza­bicyclo­[3.3.1]nonane nucleus are of great inter­est due to their presence in a wide range of naturally occurring diterpenoid/norditerpenoid alkaloids and their broad-spectrum biological activities, such as anti­microbial, analgesic, antagonistic, anti-inflammatory and local anesthetic hypotensive activity (Parthiban *et al.*, 2009 ▶; Hardick *et al.*, 1996 ▶; Jeyaraman & Avila, 1981 ▶). Hence, the synthesis of new mol­ecules with the 3-aza­bicyclo­[3.3.1]nonane nucleus and their stereochemical investigation are of inter­est in the field of medicinal chemistry. Also, the stereochemistry of such mol­ecules is a major criterium for their biological response. As a consequence, the present study was undertaken to examine the configuration and conformation of the synthesized title compound.
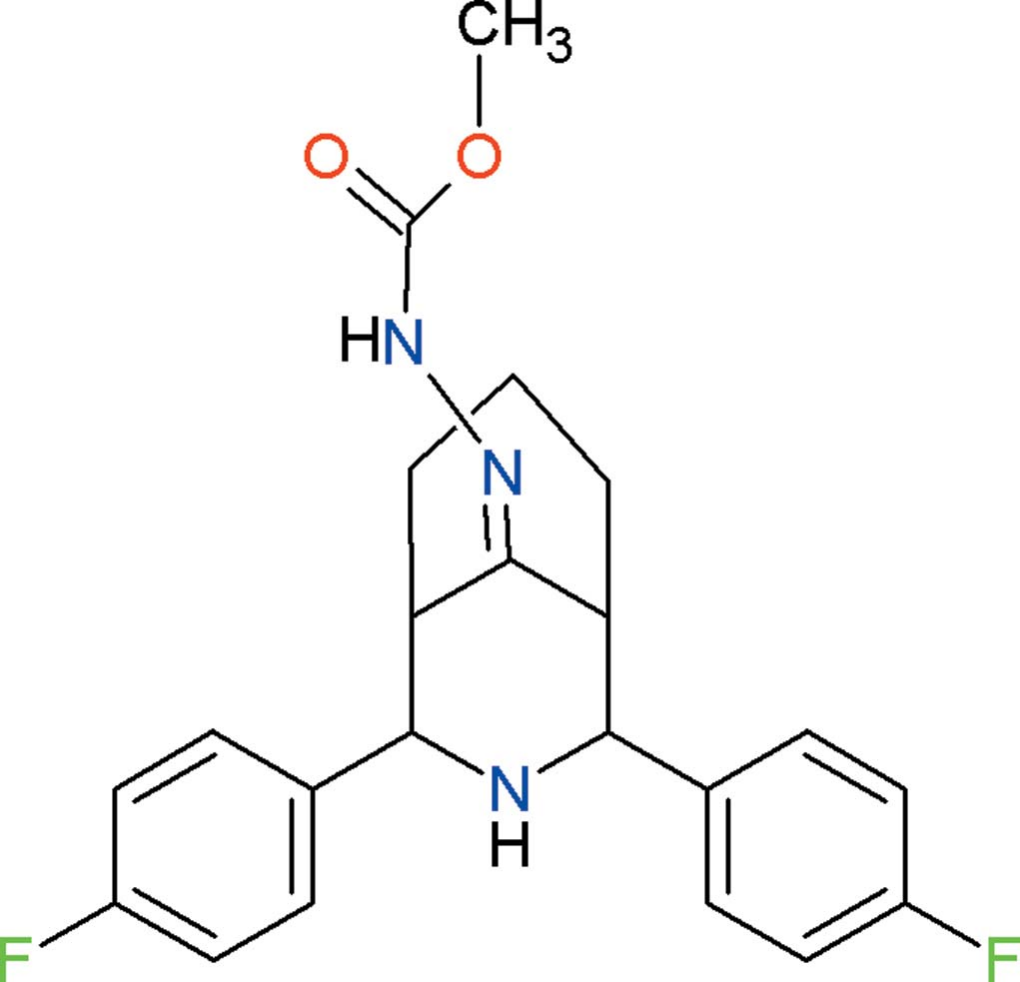


## Structural commentary   

**Figure 1:**
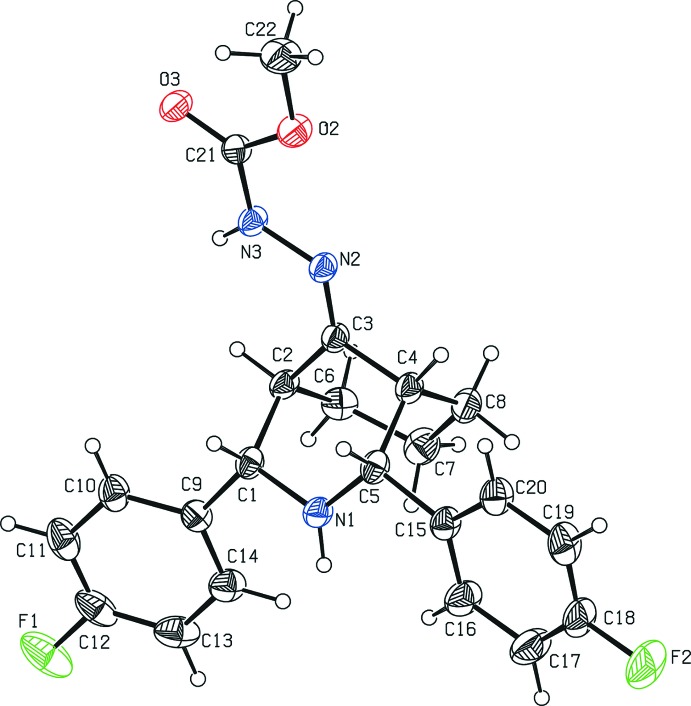
The mol­ecular structure of the title mol­ecule (I), showing the atom-labelling scheme. Displacement ellipsoids are drawn at the 30% probability level.

The mol­ecular structure of the title compound, (I), is illus­trated in Fig. 1 ▶. The bi­cyclo ring system adopts a twin-chair conformation, with puckering parameters *Q* = 0.593 (2) Å, θ = 170.8 (2)° and ϕ = 353.9 (1)° for the N1/C1–C5 piperidine ring, and *Q* = 0.546 (2) Å, θ = 10.9 (2)° and ϕ = 65.0 (1)° for the C2–C4/C6–C8) cyclo­hexane ring. The fluoro­phenyl groups on the heterocycle occupy equatorial positions and are inclined to one another by 19.4 (1)°. The geometric parameters of the title mol­ecule agree well with those reported for similar structures, for example, 2,4-bis­(4-fluoro­phen­yl)-3-aza­bicyclo­[3.3.1]nonan-9-one, (II) (Parthiban *et al.*, 2008 ▶), and 2,4-bis­(4-fluoro­phen­yl)-1,5-dimethyl-3-aza­bicyclo­[3.3.1]nonan-9-one, (III) (Rizwana Begum *et al.*, 2013 ▶).

## Supra­molecular features   

**Figure 2:**
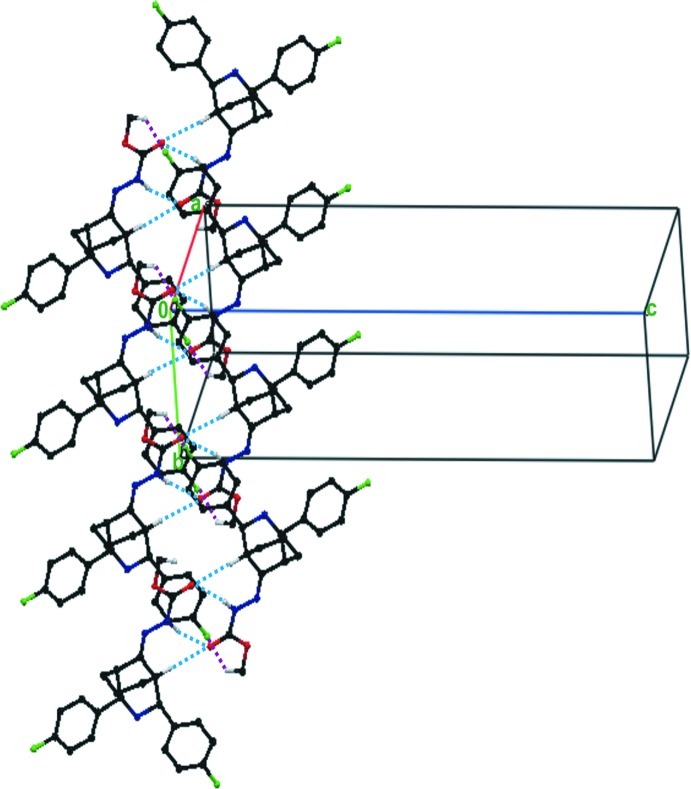
Partial view of the crystal packing of the title compound, showing the hydrogen bonds as dashed lines (see Table 1 ▶ for details). H atoms not involved in hydrogen bonding have been omitted for clarity).

In the crystal, pairs of bifurcated acceptor N3—H3⋯O3^i^ and C2—H2⋯O3^i^ (Table 1 ▶) hydrogen bonds link mol­ecules into inversion dimers, incorporating 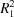
(7) and 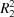
(8) ring motifs (Fig. 2 ▶). These dimers are further linked through a pair of C22—H22*C*⋯F1^ii^ (Table 1 ▶) hydrogen bonds, enclosing 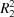
(28) ring motifs, forming supra­molecular chains along the *b*-axis direction (Fig. 2).

The NH group of the pyridine ring is not involved in hydrogen bonding, probably due to the steric hindrance of the fluoro­phenyl groups. Such a situation was reported for a similar bicyclic system substituted by di­fluoro­phenyl rings, *viz.* compound (III) (Rizwana Begum *et al.*, 2013 ▶).

## Database survey   

38 ‘hits’ for crystal structures containing the 3-aza­bicyclo­[3.3.1]nonane subunit were obtained for a search of the Cambridge Structural Database (CSD, Version 5.35, last update of February 2014; Allen, 2002 ▶). However, extending the search to allow additional substitution of 4-fluoro­phenyl groups on the bicyclic ring gave two hits, namely compounds (II) and (III) mentioned above (Section 2). Compound (III) crystallized with two independent mol­ecules (*A* and *B*) in the asymmetric unit. In all three compounds, the bi­cyclo rings have twin-chair conformations with equatorially disposed 4-fluoro­phenyl groups on the heterocycle. The fluoro­phenyl rings are oriented at an angle of 28.7 (1)° in (II), and 55.3 (1) (mol­ecule *A*) and 56.4 (1)° (mol­ecule *B*) for (III), compared to 19.4 (1)° in the title compound, (I).

## Synthesis and crystallization   

A mixture of 2,4-diphenyl-3-aza­bicyclo­[3.3.1]nonan-9-one (0.1 mmol), methyl hydrazine­carboxyl­ate (1.5 mmol) in an ethanol–chloro­form (1:1 *v*/*v*) medium, with the addition of few drops of acetic acid, was stirred for 10–12 h. After completion of the reaction a solid mass was formed. The precipitate was filtered off and washed with an ethanol–water mixture. The crude product was then recrystallized from ethanol–chloro­form to obtain colourless diffraction-quality crystals of title compound.

## Refinement   

Crystal data, data collection and structure refinement details are summarized in Table 2 ▶. All H atoms were positioned geometrically and constrained to ride on their parent atom, with N—H = 0.86 Å and C—H = 0.93–0.97 Å, and with *U*_iso_(H) = 1.5*U*_eq_(C) for methyl H atoms and 1.2*U*_eq_(N,C) for all other H atoms.

## Supplementary Material

Crystal structure: contains datablock(s) global, I. DOI: 10.1107/S1600536814018935/su2773sup1.cif

Structure factors: contains datablock(s) I. DOI: 10.1107/S1600536814018935/su2773Isup2.hkl

Supporting information file. DOI: 10.1107/S1600536814018935/su2773Isup3.cml

CCDC reference: 1020373

Additional supporting information:  crystallographic information; 3D view; checkCIF report

## Figures and Tables

**Table 1:** Hydrogen-bond geometry (Å, °)

*D*—H⋯*A*	*D*—H	H⋯*A*	*D*⋯*A*	*D*—H⋯*A*
N3—H3⋯O3^i^	0.86	2.12	2.930 (2)	157
C2—H2⋯O3^i^	0.98	2.49	3.442 (2)	163
C22—H22*C*⋯F1^ii^	0.96	2.49	3.243 (3)	136

Symmetry codes: (i) 
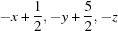
; (ii) 
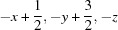
.

**Table 2:** Experimental details

Crystal data	
Chemical formula	C_22_H_23_F_2_N_3_O_2_
*M*_r_	399.43
Crystal system, space group	Monoclinic, *C*2/*c*
Temperature (K)	293
*a*, *b*, *c* (Å)	19.751 (6), 7.087 (2), 28.492 (9)
β (°)	102.997 (4)
*V* (Å^3^)	3886 (2)
*Z*	8
Radiation type	Mo *K*α
μ (mm^−1^)	0.10
Crystal size (mm)	0.38 × 0.36 × 0.34

Data collection	
Diffractometer	Bruker SMART CCD area detector
Absorption correction	Multi-scan (*SADABS*; Sheldrick, 1996 ▶)
*T*_min_, *T*_max_	0.962, 0.966
No. of measured, independent and observed [*I* > 2σ(*I*)] reflections	18613, 3793, 2872
*R*_int_	0.030
(sin θ/λ)_max_ (Å^−1^)	0.618

Refinement	
*R*[*F* ^2^ > 2σ(*F* ^2^)], *wR*(*F* ^2^), *S*	0.050, 0.147, 1.04
No. of reflections	3793
No. of parameters	263
H-atom treatment	H-atom parameters constrained
Δρ_max_, Δρ_min_ (e Å^−3^)	0.52, −0.44

Computer programs: *SMART* and *SAINT* (Bruker, 2002 ▶), *SHELXS97* and *SHELXL97* (Sheldrick, 2008 ▶), *ORTEP-3 for Windows* (Farrugia (2012 ▶) and *PLATON* (Spek, 2009 ▶).

## supplementary crystallographic information

### Crystal data

**Table:**

C_22_H_23_F_2_N_3_O_2_	*F*(000) = 1680
*M**_r_* = 399.43	*D*_x_ = 1.365 Mg m^−^^3^
Monoclinic, *C*2/*c*	Mo *K*α radiation, λ = 0.71073 Å
Hall symbol: -C 2yc	Cell parameters from 3821 reflections
*a* = 19.751 (6) Å	θ = 1.5–26.1°
*b* = 7.087 (2) Å	µ = 0.10 mm^−^^1^
*c* = 28.492 (9) Å	*T* = 293 K
β = 102.997 (4)°	Needle, colourless
*V* = 3886 (2) Å^3^	0.38 × 0.36 × 0.34 mm
*Z* = 8	

### Data collection

**Table:**

Bruker SMART CCD area-detector diffractometer	3793 independent reflections
Radiation source: fine-focus sealed tube	2872 reflections with *I* > 2σ(*I*)
Graphite monochromator	*R*_int_ = 0.030
φ and ω scans	θ_max_ = 26.1°, θ_min_ = 1.5°
Absorption correction: multi-scan (*SADABS*; Sheldrick, 1996)	*h* = −24→24
*T*_min_ = 0.962, *T*_max_ = 0.966	*k* = −8→8
18613 measured reflections	*l* = −35→35

### Refinement

**Table:**

Refinement on *F*^2^	Primary atom site location: structure-invariant direct methods
Least-squares matrix: full	Secondary atom site location: difference Fourier map
*R*[*F*^2^ > 2σ(*F*^2^)] = 0.050	Hydrogen site location: inferred from neighbouring sites
*wR*(*F*^2^) = 0.147	H-atom parameters constrained
*S* = 1.04	*w* = 1/[σ^2^(*F*_o_^2^) + (0.0752*P*)^2^ + 2.7221*P*] where *P* = (*F*_o_^2^ + 2*F*_c_^2^)/3
3793 reflections	(Δ/σ)_max_ < 0.001
263 parameters	Δρ_max_ = 0.52 e Å^−^^3^
0 restraints	Δρ_min_ = −0.44 e Å^−^^3^

### Special details

**Table:**

Geometry. All esds (except the esd in the dihedral angle between two l.s. planes) are estimated using the full covariance matrix. The cell esds are taken into account individually in the estimation of esds in distances, angles and torsion angles; correlations between esds in cell parameters are only used when they are defined by crystal symmetry. An approximate (isotropic) treatment of cell esds is used for estimating esds involving l.s. planes.
Refinement. Refinement of F^2^ against ALL reflections. The weighted R-factor wR and goodness of fit S are based on F^2^, conventional R-factors R are based on F, with F set to zero for negative F^2^. The threshold expression of F^2^ > 2sigma(F^2^) is used only for calculating R-factors(gt) etc. and is not relevant to the choice of reflections for refinement. R-factors based on F^2^ are statistically about twice as large as those based on F, and R- factors based on ALL data will be even larger.

### Fractional atomic coordinates and isotropic or equivalent isotropic displacement parameters (Å^2^)

**Table:**

	*x*	*y*	*z*	*U*_iso_*/*U*_eq_	
C1	0.34340 (10)	0.7153 (3)	0.09187 (6)	0.0395 (4)	
H1	0.2935	0.7216	0.0775	0.047*	
C2	0.37276 (10)	0.9185 (3)	0.09443 (6)	0.0397 (4)	
H2	0.3614	0.9740	0.0621	0.048*	
C3	0.33596 (9)	1.0266 (3)	0.12629 (6)	0.0375 (4)	
C4	0.35286 (10)	0.9504 (3)	0.17646 (6)	0.0418 (4)	
H4	0.3280	1.0258	0.1960	0.050*	
C5	0.32457 (10)	0.7461 (3)	0.17394 (6)	0.0411 (4)	
H5	0.2740	0.7514	0.1625	0.049*	
C6	0.45136 (10)	0.9334 (3)	0.11470 (7)	0.0488 (5)	
H6A	0.4747	0.8521	0.0960	0.059*	
H6B	0.4658	1.0619	0.1105	0.059*	
C7	0.47506 (11)	0.8806 (4)	0.16730 (8)	0.0568 (6)	
H7A	0.4737	0.7445	0.1704	0.068*	
H7B	0.5228	0.9211	0.1790	0.068*	
C8	0.43043 (11)	0.9689 (3)	0.19781 (7)	0.0506 (5)	
H8A	0.4421	1.1016	0.2021	0.061*	
H8B	0.4411	0.9100	0.2294	0.061*	
C9	0.37645 (10)	0.5907 (3)	0.06043 (7)	0.0415 (4)	
C10	0.35221 (11)	0.5986 (3)	0.01104 (7)	0.0500 (5)	
H10	0.3158	0.6797	−0.0020	0.060*	
C11	0.38109 (13)	0.4882 (4)	−0.01910 (8)	0.0609 (6)	
H11	0.3648	0.4941	−0.0523	0.073*	
C12	0.43378 (14)	0.3710 (3)	0.00080 (9)	0.0622 (7)	
C13	0.45997 (13)	0.3609 (3)	0.04883 (9)	0.0631 (6)	
H13	0.4969	0.2808	0.0614	0.076*	
C14	0.43080 (12)	0.4716 (3)	0.07882 (8)	0.0530 (5)	
H14	0.4482	0.4656	0.1119	0.064*	
C15	0.34057 (10)	0.6549 (3)	0.22311 (6)	0.0435 (5)	
C16	0.38849 (13)	0.5154 (3)	0.23568 (8)	0.0603 (6)	
H16	0.4112	0.4693	0.2128	0.072*	
C17	0.40412 (15)	0.4407 (4)	0.28160 (9)	0.0718 (7)	
H17	0.4368	0.3449	0.2897	0.086*	
C18	0.37098 (14)	0.5097 (4)	0.31434 (8)	0.0628 (6)	
C19	0.32234 (13)	0.6474 (4)	0.30379 (7)	0.0618 (6)	
H19	0.3001	0.6920	0.3271	0.074*	
C20	0.30660 (12)	0.7197 (3)	0.25771 (7)	0.0530 (5)	
H20	0.2728	0.8129	0.2497	0.064*	
C21	0.22233 (9)	1.3588 (3)	0.05966 (6)	0.0383 (4)	
C22	0.14426 (13)	1.5496 (4)	0.08890 (9)	0.0650 (6)	
H22A	0.1636	1.6721	0.0859	0.098*	
H22B	0.1214	1.5500	0.1153	0.098*	
H22C	0.1113	1.5189	0.0597	0.098*	
N1	0.35236 (8)	0.6352 (2)	0.13996 (5)	0.0416 (4)	
H1A	0.3728	0.5289	0.1477	0.050*	
N2	0.29174 (8)	1.1591 (2)	0.11708 (5)	0.0384 (4)	
N3	0.27210 (8)	1.2269 (2)	0.07053 (5)	0.0396 (4)	
H3	0.2917	1.1846	0.0486	0.048*	
O2	0.19861 (7)	1.4123 (2)	0.09762 (5)	0.0516 (4)	
O3	0.20192 (7)	1.4228 (2)	0.01966 (5)	0.0487 (4)	
F1	0.46171 (10)	0.2594 (2)	−0.02847 (6)	0.0940 (6)	
F2	0.38685 (10)	0.4378 (3)	0.35949 (5)	0.0986 (6)	

### Atomic displacement parameters (Å^2^)

**Table:**

	*U*^11^	*U*^22^	*U*^33^	*U*^12^	*U*^13^	*U*^23^
C1	0.0405 (10)	0.0478 (11)	0.0313 (9)	0.0021 (8)	0.0106 (7)	0.0020 (8)
C2	0.0476 (10)	0.0434 (10)	0.0304 (9)	0.0083 (8)	0.0135 (7)	0.0069 (7)
C3	0.0404 (10)	0.0407 (10)	0.0313 (9)	0.0023 (8)	0.0076 (7)	0.0024 (7)
C4	0.0489 (11)	0.0472 (11)	0.0297 (9)	0.0082 (9)	0.0099 (8)	0.0010 (8)
C5	0.0401 (10)	0.0529 (11)	0.0321 (9)	0.0018 (9)	0.0118 (7)	0.0029 (8)
C6	0.0480 (11)	0.0475 (11)	0.0548 (12)	0.0002 (9)	0.0196 (9)	0.0048 (9)
C7	0.0443 (11)	0.0638 (14)	0.0594 (13)	−0.0012 (10)	0.0058 (10)	0.0053 (11)
C8	0.0551 (12)	0.0540 (12)	0.0389 (10)	−0.0035 (10)	0.0025 (9)	0.0043 (9)
C9	0.0470 (11)	0.0421 (10)	0.0393 (10)	−0.0047 (9)	0.0176 (8)	−0.0004 (8)
C10	0.0563 (12)	0.0567 (13)	0.0398 (10)	−0.0074 (10)	0.0166 (9)	−0.0040 (9)
C11	0.0782 (16)	0.0654 (15)	0.0465 (12)	−0.0200 (13)	0.0297 (11)	−0.0146 (11)
C12	0.0825 (17)	0.0447 (12)	0.0751 (16)	−0.0148 (12)	0.0510 (14)	−0.0160 (11)
C13	0.0748 (16)	0.0466 (12)	0.0798 (17)	0.0107 (11)	0.0427 (13)	0.0097 (11)
C14	0.0636 (13)	0.0503 (12)	0.0507 (12)	0.0054 (10)	0.0247 (10)	0.0076 (9)
C15	0.0483 (11)	0.0491 (11)	0.0357 (10)	−0.0036 (9)	0.0148 (8)	0.0044 (8)
C16	0.0775 (16)	0.0636 (14)	0.0453 (12)	0.0138 (12)	0.0253 (11)	0.0119 (10)
C17	0.0903 (19)	0.0704 (17)	0.0563 (14)	0.0167 (14)	0.0196 (13)	0.0236 (12)
C18	0.0850 (17)	0.0676 (15)	0.0365 (11)	−0.0139 (13)	0.0149 (11)	0.0140 (10)
C19	0.0779 (16)	0.0761 (16)	0.0387 (11)	−0.0159 (13)	0.0286 (11)	−0.0025 (11)
C20	0.0594 (13)	0.0634 (14)	0.0411 (11)	−0.0017 (11)	0.0217 (9)	0.0018 (10)
C21	0.0400 (10)	0.0384 (10)	0.0379 (10)	0.0013 (8)	0.0114 (8)	0.0006 (8)
C22	0.0655 (15)	0.0639 (15)	0.0700 (15)	0.0241 (12)	0.0245 (12)	−0.0003 (12)
N1	0.0524 (9)	0.0429 (9)	0.0319 (8)	0.0040 (7)	0.0146 (7)	0.0046 (6)
N2	0.0431 (9)	0.0424 (9)	0.0303 (8)	0.0052 (7)	0.0093 (6)	0.0031 (6)
N3	0.0439 (8)	0.0449 (9)	0.0317 (8)	0.0089 (7)	0.0122 (6)	0.0041 (6)
O2	0.0583 (9)	0.0564 (9)	0.0440 (8)	0.0180 (7)	0.0196 (6)	0.0038 (6)
O3	0.0555 (8)	0.0514 (8)	0.0389 (7)	0.0143 (7)	0.0099 (6)	0.0087 (6)
F1	0.1259 (14)	0.0680 (10)	0.1142 (13)	−0.0134 (9)	0.0820 (11)	−0.0356 (9)
F2	0.1409 (15)	0.1101 (13)	0.0446 (8)	−0.0095 (11)	0.0204 (9)	0.0314 (8)

### Geometric parameters (Å, º)

**Table:**

C1—N1	1.457 (2)	C11—H11	0.9300
C1—C9	1.507 (3)	C12—C13	1.352 (3)
C1—C2	1.548 (3)	C12—F1	1.354 (2)
C1—H1	0.9800	C13—C14	1.378 (3)
C2—C3	1.495 (2)	C13—H13	0.9300
C2—C6	1.533 (3)	C14—H14	0.9300
C2—H2	0.9800	C15—C16	1.360 (3)
C3—N2	1.269 (2)	C15—C20	1.389 (3)
C3—C4	1.494 (2)	C16—C17	1.381 (3)
C4—C8	1.522 (3)	C16—H16	0.9300
C4—C5	1.548 (3)	C17—C18	1.346 (4)
C4—H4	0.9800	C17—H17	0.9300
C5—N1	1.447 (2)	C18—F2	1.353 (2)
C5—C15	1.510 (3)	C18—C19	1.355 (4)
C5—H5	0.9800	C19—C20	1.378 (3)
C6—C7	1.513 (3)	C19—H19	0.9300
C6—H6A	0.9700	C20—H20	0.9300
C6—H6B	0.9700	C21—O3	1.208 (2)
C7—C8	1.506 (3)	C21—O2	1.327 (2)
C7—H7A	0.9700	C21—N3	1.342 (2)
C7—H7B	0.9700	C22—O2	1.428 (3)
C8—H8A	0.9700	C22—H22A	0.9600
C8—H8B	0.9700	C22—H22B	0.9600
C9—C14	1.373 (3)	C22—H22C	0.9600
C9—C10	1.382 (3)	N1—H1A	0.8600
C10—C11	1.376 (3)	N2—N3	1.3814 (19)
C10—H10	0.9300	N3—H3	0.8600
C11—C12	1.352 (4)		
			
N1—C1—C9	110.73 (15)	C11—C10—H10	119.5
N1—C1—C2	110.67 (15)	C9—C10—H10	119.5
C9—C1—C2	111.42 (15)	C12—C11—C10	118.4 (2)
N1—C1—H1	108.0	C12—C11—H11	120.8
C9—C1—H1	108.0	C10—C11—H11	120.8
C2—C1—H1	108.0	C13—C12—C11	122.7 (2)
C3—C2—C6	108.99 (16)	C13—C12—F1	118.4 (2)
C3—C2—C1	106.09 (14)	C11—C12—F1	118.8 (2)
C6—C2—C1	114.73 (16)	C12—C13—C14	118.6 (2)
C3—C2—H2	109.0	C12—C13—H13	120.7
C6—C2—H2	109.0	C14—C13—H13	120.7
C1—C2—H2	109.0	C9—C14—C13	120.9 (2)
N2—C3—C4	117.43 (16)	C9—C14—H14	119.6
N2—C3—C2	131.26 (16)	C13—C14—H14	119.6
C4—C3—C2	111.18 (15)	C16—C15—C20	118.16 (18)
C3—C4—C8	109.85 (16)	C16—C15—C5	122.92 (17)
C3—C4—C5	107.04 (15)	C20—C15—C5	118.90 (19)
C8—C4—C5	114.81 (16)	C15—C16—C17	121.5 (2)
C3—C4—H4	108.3	C15—C16—H16	119.2
C8—C4—H4	108.3	C17—C16—H16	119.2
C5—C4—H4	108.3	C18—C17—C16	118.5 (2)
N1—C5—C15	110.89 (16)	C18—C17—H17	120.7
N1—C5—C4	110.59 (15)	C16—C17—H17	120.7
C15—C5—C4	111.04 (15)	C17—C18—F2	118.5 (3)
N1—C5—H5	108.1	C17—C18—C19	122.6 (2)
C15—C5—H5	108.1	F2—C18—C19	118.9 (2)
C4—C5—H5	108.1	C18—C19—C20	118.4 (2)
C7—C6—C2	114.68 (16)	C18—C19—H19	120.8
C7—C6—H6A	108.6	C20—C19—H19	120.8
C2—C6—H6A	108.6	C19—C20—C15	120.8 (2)
C7—C6—H6B	108.6	C19—C20—H20	119.6
C2—C6—H6B	108.6	C15—C20—H20	119.6
H6A—C6—H6B	107.6	O3—C21—O2	123.87 (17)
C8—C7—C6	112.23 (18)	O3—C21—N3	123.44 (17)
C8—C7—H7A	109.2	O2—C21—N3	112.68 (15)
C6—C7—H7A	109.2	O2—C22—H22A	109.5
C8—C7—H7B	109.2	O2—C22—H22B	109.5
C6—C7—H7B	109.2	H22A—C22—H22B	109.5
H7A—C7—H7B	107.9	O2—C22—H22C	109.5
C7—C8—C4	113.64 (17)	H22A—C22—H22C	109.5
C7—C8—H8A	108.8	H22B—C22—H22C	109.5
C4—C8—H8A	108.8	C5—N1—C1	115.71 (15)
C7—C8—H8B	108.8	C5—N1—H1A	122.1
C4—C8—H8B	108.8	C1—N1—H1A	122.1
H8A—C8—H8B	107.7	C3—N2—N3	119.17 (15)
C14—C9—C10	118.38 (19)	C21—N3—N2	119.81 (14)
C14—C9—C1	122.62 (17)	C21—N3—H3	120.1
C10—C9—C1	118.98 (18)	N2—N3—H3	120.1
C11—C10—C9	121.0 (2)	C21—O2—C22	116.23 (16)
			
N1—C1—C2—C3	−56.46 (19)	C11—C12—C13—C14	1.4 (4)
C9—C1—C2—C3	179.87 (15)	F1—C12—C13—C14	−178.8 (2)
N1—C1—C2—C6	63.9 (2)	C10—C9—C14—C13	−0.8 (3)
C9—C1—C2—C6	−59.8 (2)	C1—C9—C14—C13	−179.51 (19)
C6—C2—C3—N2	124.6 (2)	C12—C13—C14—C9	−0.3 (3)
C1—C2—C3—N2	−111.4 (2)	N1—C5—C15—C16	14.8 (3)
C6—C2—C3—C4	−59.9 (2)	C4—C5—C15—C16	−108.6 (2)
C1—C2—C3—C4	64.16 (19)	N1—C5—C15—C20	−167.30 (17)
N2—C3—C4—C8	−122.34 (19)	C4—C5—C15—C20	69.3 (2)
C2—C3—C4—C8	61.4 (2)	C20—C15—C16—C17	−0.9 (4)
N2—C3—C4—C5	112.41 (19)	C5—C15—C16—C17	177.1 (2)
C2—C3—C4—C5	−63.81 (19)	C15—C16—C17—C18	−0.3 (4)
C3—C4—C5—N1	55.25 (19)	C16—C17—C18—F2	−179.3 (2)
C8—C4—C5—N1	−67.0 (2)	C16—C17—C18—C19	0.9 (4)
C3—C4—C5—C15	178.80 (15)	C17—C18—C19—C20	−0.3 (4)
C8—C4—C5—C15	56.6 (2)	F2—C18—C19—C20	180.0 (2)
C3—C2—C6—C7	52.2 (2)	C18—C19—C20—C15	−1.0 (4)
C1—C2—C6—C7	−66.5 (2)	C16—C15—C20—C19	1.5 (3)
C2—C6—C7—C8	−45.8 (3)	C5—C15—C20—C19	−176.5 (2)
C6—C7—C8—C4	46.3 (3)	C15—C5—N1—C1	−176.69 (15)
C3—C4—C8—C7	−54.2 (2)	C4—C5—N1—C1	−53.1 (2)
C5—C4—C8—C7	66.5 (2)	C9—C1—N1—C5	178.09 (15)
N1—C1—C9—C14	−26.0 (3)	C2—C1—N1—C5	54.0 (2)
C2—C1—C9—C14	97.7 (2)	C4—C3—N2—N3	−176.76 (16)
N1—C1—C9—C10	155.29 (17)	C2—C3—N2—N3	−1.5 (3)
C2—C1—C9—C10	−81.1 (2)	O3—C21—N3—N2	−179.05 (17)
C14—C9—C10—C11	0.8 (3)	O2—C21—N3—N2	1.6 (2)
C1—C9—C10—C11	179.62 (18)	C3—N2—N3—C21	175.91 (17)
C9—C10—C11—C12	0.2 (3)	O3—C21—O2—C22	2.0 (3)
C10—C11—C12—C13	−1.4 (3)	N3—C21—O2—C22	−178.61 (18)
C10—C11—C12—F1	178.83 (19)		

### Hydrogen-bond geometry (Å, º)

**Table:**

*D*—H···*A*	*D*—H	H···*A*	*D*···*A*	*D*—H···*A*
N3—H3···O3^i^	0.86	2.12	2.930 (2)	157
C2—H2···O3^i^	0.98	2.49	3.442 (2)	163
C22—H22*C*···F1^ii^	0.96	2.49	3.243 (3)	136

Symmetry codes: (i) −*x*+1/2, −*y*+5/2, −*z*; (ii) −*x*+1/2, −*y*+3/2, −*z*.
